# Targeting Mitochondrial Dysfunction and Reactive Oxygen Species for Neurodegenerative Disease Treatment

**DOI:** 10.3390/ijms25147952

**Published:** 2024-07-21

**Authors:** Eui-Hwan Choi, Mi-Hye Kim, Sun-Ji Park

**Affiliations:** New Drug Development Center, Daegu-Gyeongbuk Medical Innovation Foundation (DGMIF), Daegu 41061, Republic of Korea; priestbh2@gmail.com (E.-H.C.); mihyekk@kmedihub.re.kr (M.-H.K.)

**Keywords:** mitochondrial dysfunction, reactive oxygen species, Alzheimer’s disease, Parkinson’s disease

## Abstract

Alzheimer’s disease (AD) and Parkinson’s disease (PD) are the most common neurodegenerative diseases, and they affect millions of people worldwide, particularly older individuals. Therefore, there is a clear need to develop novel drug targets for the treatment of age-related neurodegenerative diseases. Emerging evidence suggests that mitochondrial dysfunction and reactive oxygen species (ROS) generation play central roles in the onset and progression of neurodegenerative diseases. Mitochondria are key regulators of respiratory function, cellular energy adenosine triphosphate production, and the maintenance of cellular redox homeostasis, which are essential for cell survival. Mitochondrial morphology and function are tightly regulated by maintaining a balance among mitochondrial fission, fusion, biogenesis, and mitophagy. In this review, we provide an overview of the main functions of mitochondria, with a focus on recent progress highlighting the critical role of ROS−induced oxidative stress, dysregulated mitochondrial dynamics, mitochondrial apoptosis, mitochondria-associated inflammation, and impaired mitochondrial function in the pathogenesis of age-related neurodegenerative diseases, such as AD and PD. We also discuss the potential of mitochondrial fusion and biogenesis enhancers, mitochondrial fission inhibitors, and mitochondria-targeted antioxidants as novel drugs for the treatment of these diseases.

## 1. Introduction

Mitochondria play many essential roles in cells, including regulating the oxidative phosphorylation system (OXPHOS) and carbon metabolism; producing adenosine triphosphate (ATP) for cellular energy; and controlling intracellular signaling molecules, such as reactive oxygen species (ROS) and Ca^2+^ for cell function, which are also involved in several other key processes that determine cell survival [1,2,3,4]. Mitochondrial homeostasis is tightly regulated by maintaining a balance among mitochondrial fission, fusion, biogenesis, and mitophagy, which is referred to as mitochondrial dynamics [5,6,7]. Aging; genetic mutations; and environmental factors, such as toxic chemicals and smoking, are associated with the impairment of mitochondrial function and dynamics and the induction of oxidative stress [3,8]. Furthermore, mitochondrial dysfunction and increased oxidative stress have been found to contribute to many common disorders, including chronic kidney disease, metabolic disease, and neurodegenerative disease [9,10,11,12].

Alzheimer’s disease (AD), Parkinson’s disease (PD), amyotrophic lateral sclerosis, and Huntington’s disease are four representative neurodegenerative disorders [10]. Among these, AD and PD are the most common age-related neurodegenerative diseases that affect millions of people worldwide and are the leading causes of morbidity and cognitive decline in the elderly population [9,11]. Currently, there are no effective treatments for AD or PD. Thus, there is a clear need to identify novel drug targets for the treatment of age-related neurodegenerative diseases [10,13]. Emerging evidence indicates that impaired mitochondrial function and oxidative stress induced by excessive ROS production are implicated in the onset and progression of neurodegenerative diseases [9,11,13,14]. Therefore, we provide an overview of mitochondrial function and morphological changes and discuss several recent studies that highlight the critical role of ROS−induced oxidative stress, dysregulated mitochondrial dynamics, mitochondrial apoptosis, mitochondria-associated inflammation, and impaired mitochondrial function in the pathogenesis of AD and PD. We also suggest mitochondria-targeted drugs, including mitochondrial fusion and biogenesis enhancers, mitochondrial fission inhibitors, and mitochondria-targeted antioxidants as novel therapeutic drugs for treating neurodegenerative diseases.

## 2. Mitochondrial Function and Dysfunction

### 2.1. Mitochondrial Energy Production and Oxidative Stress

**Figure 1:**
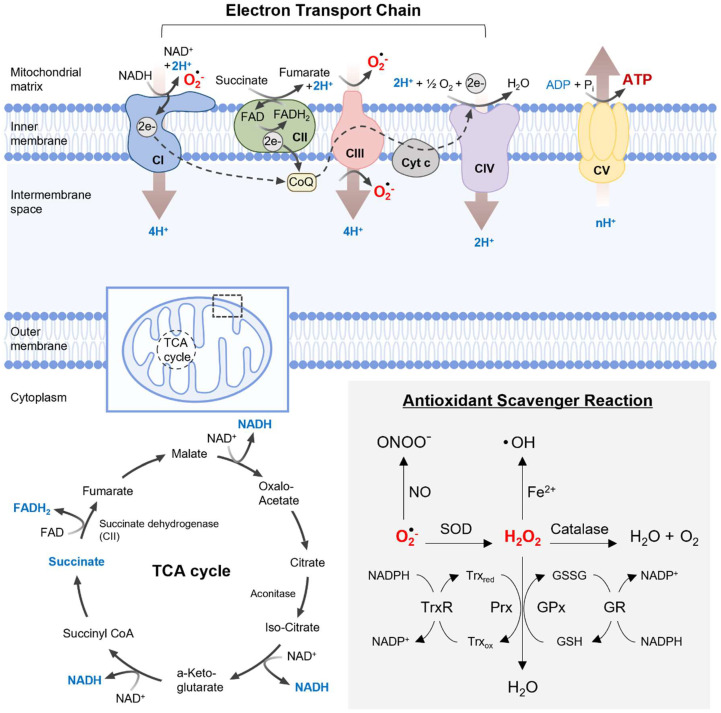
Illustrates the ROS generation in the mitochondrion. The mitochondrial ETC consists of five multi-subunit enzyme complexes located in the IMM. Electrons donated by NADH and FADH_2_ during the TCA cycle are shuttled to ETC components, initiating at complex I (NADH ubiquinone reductase) or complex II (succinate dehydrogenase), and then sequentially passing through complex III (ubiquinol-cytochrome c reductase) and complex IV (cytochrome c oxidase), finally reaching complex V (F0F1 ATP synthase), where they are finally transferred to oxygen. This electron transfer process is closely associated with proton transport across the inner membrane, establishing an electrochemical gradient crucial for ATP generation. Under normal conditions, mitochondria efficiently metabolize oxygen, producing ROS such as O_2_^•−^, which serves as the primary ROS. O_2_^•−^ is then converted to H_2_O_2_ by SOD within the mitochondria. Subsequently, H_2_O_2_ is further metabolized to water by enzymes such as GPx−GR, Prx−Trx−TrxR, or catalase. However, excessive ROS production can lead to oxidative damage to proteins, lipids, and mtDNA.

Mitochondria are membrane-bound cytosolic organelles that generate the chemical energy required to power the cellular biochemical reactions [1,2]. They play an essential role in producing energy-rich molecules and breaking down nutrients in cells [2]. Most biochemical reactions involved in the cell cycle occur in the mitochondria [8]. In eukaryotic cells, mitochondria produce energy in the form of ATP through nutrient oxidative metabolism via the following two major processes: (1) NADH or FADH_2_ oxidation generated during the tricarboxylic acid (TCA) cycle or glycolysis and (2) oxidative phosphorylation to produce ATP [3]. Various transcription factors regulate mitochondrial respiration. Mitochondria have 800−1600 copies of their own DNA (known as mitochondrial DNA or mtDNA), which are genetically inherited and packaged into distinct mtDNA-protein structures referred to as nucleoids [15,16]. Nucleoids present a dynamic form that undergoes fission/fusion and they are distributed throughout the mitochondrial matrix [7]. However, only a few nucleoids that carry the mitochondrial OXPHOS system are located close to the cristae. The mitochondrial structure has the following three compartments: the outer mitochondrial membrane (OMM), inner mitochondrial membrane (IMM), and matrix [1,17]. These compartments have distinct roles and corresponding protein components. The OMM and intermembrane space are relatively more permeable than the IMM [18]. In contrast, the IMM has much more restricted permeability and contains enzymes involved in the electron transport chain (ETC) and ATP generation [19,20]. The reactions of oxidation or reduction along the components of the ETC produce a proton gradient, which is used by ATP synthase to phosphorylate ADP, thereby producing ATP [19]. To increase the capacity of mitochondria to synthesize ATP, the inner membranes are folded to form cristae. The folded inner membrane leads to a much higher number of ETC enzymes and allows ATP to be packed into the mitochondria [20] (Figure 1).

The OXPHOS-ETC of the IMM is composed of five multi-subunit protein complexes named complex I–complex V [3,21]. The electrons donated by NADH and FADH2 under the TCA cycle are accepted and transferred to ETC components in complexes I (NADH ubiquinone reductase, NADH dehydrogenase), II (succinate ubiquinone oxidoreductase, succinate dehydrogenase), III (ubiquinol−cytochrome *c* oxidoreductase, cytochrome *bc*1 complex), and IV (cytochrome *c* oxidase) [22]. Finally, OXPHOS−ETC is accomplished by complex V (F01F1 ATP synthase). This electron flow is coupled with the production of a proton gradient across the inner membrane, and energy stored in the electrochemical gradient is used by complex V to generate ATP [21]. As a central byproduct of ATP generation, mitochondria generate ROS and metabolize oxygen (O_2_). However, electron flow through the ETC is an unstable process, in which 0.1~5% of the oxygen used by the mitochondria is imperfectly reduced [4,8,23]. This generates the highly reactive ROS, superoxide anion (O_2_^•−^), within cells. Excess O_2_^•−^ can interact with diverse chemicals and/or compounds and produces secondary ROS, including the hydroxyl radical (OH^•^) and hydrogen peroxide(H_2_O_2_). OH^•^ is an extremely toxic ROS with high reactivity and a short half-life. It is generated from H_2_O_2_ in the presence of iron or copper. Increased mitochondrial ROS levels can cause the collapse of the mitochondrial membrane potential (MMP, ΔΨ m), which in turn, produces more ROS and inhibits ATP generation and essential mitochondrial functions. Excessive production of ROS leads to oxidative damage to mitochondria and other cellular organelles, as well as to cellular components, such as proteins, lipids, and DNA [24,25]. In addition, mitochondria produce reactive nitrogen species (RNS), such as nitric oxide (NO), through mitochondrial NO synthase (mtNOS). The reaction of NO with O_2_^•−^ generates peroxynitrite (ONOO^-^), which controls signaling molecules and modifies protein functions through the nitration of tyrosine residues and the oxidation of thiol groups of cysteine residues. Similar to ROS, RNS are highly toxic and can cause protein dysfunction and induce oxidative/nitrative stress [24]. Cells have various strategies to prevent oxidative stress by directly diminishing the generation of ROS/RNS and scavenging the ROS/RNS through an array of antioxidant systems, including both enzymatic and non-enzymatic antioxidants, such as vitamins and flavonoids [4,24]. Therefore, mitochondria-derived ROS/RNS are key regulators of mitochondrial function and play a central role in determining cell survival.

### 2.2. Antioxidant Defense System

Cells use several defense systems to help prevent oxidative stress caused by the overproduction of ROS. Superoxide dismutase (SOD), glutathione peroxidase (GPx), peroxiredoxin (Prx), thioredoxin (Trx), and catalase (CAT) are antioxidants involved in the enzymatic defense system [26]. NAPDH oxidases and the mitochondrial ETC are the major sources of O_2_^•−^. SOD quickly converts O_2_^•−^ to H_2_O_2_, and then H_2_O_2_ can diffuse across the membrane. H_2_O_2_ can be converted to H_2_O by mitochondrial and cytosolic GPx, Prx, and CAT. Increased intracellular H_2_O_2_ is reduced by the thiol–disulfide exchange reactions of GPx and Prx. The oxidized forms of GPx and Prx are reduced by the activity of glutathione reductase (GR) and Trx−Trx reductase (TrxR), respectively. CAT also neutralizes H_2_O_2_ to form H_2_O and oxygen. The cooperation of these antioxidant enzymes protects cells against oxidative damage and prevents pathological changes [23,26]. If not reduced by antioxidant enzymes, most types of ROS have lethal toxicity to cells and cellular organelles, particularly mitochondria. This diminishes enzyme function in the respiratory cycle and ultimately causes mitochondrial dysfunction; reduces the generation of new mitochondrial material from pre-existing mitochondria; and leads to a broad range of pathological conditions, such as various genetic diseases, aging, and neurodegenerative disorders [14,27] (Figure 1).

## 3. Mitochondrial Dynamics

Mitochondrial fusion occurs when two adjacent mitochondria merge, whereas fission involves the separation of one mitochondrion into two. These processes constantly counterbalance each other, and the inactivation of one leads to unopposed action by the other, thereby influencing mitochondrial structure and function.

### 3.1. Mitochondrial Fusion

**Figure 2:**
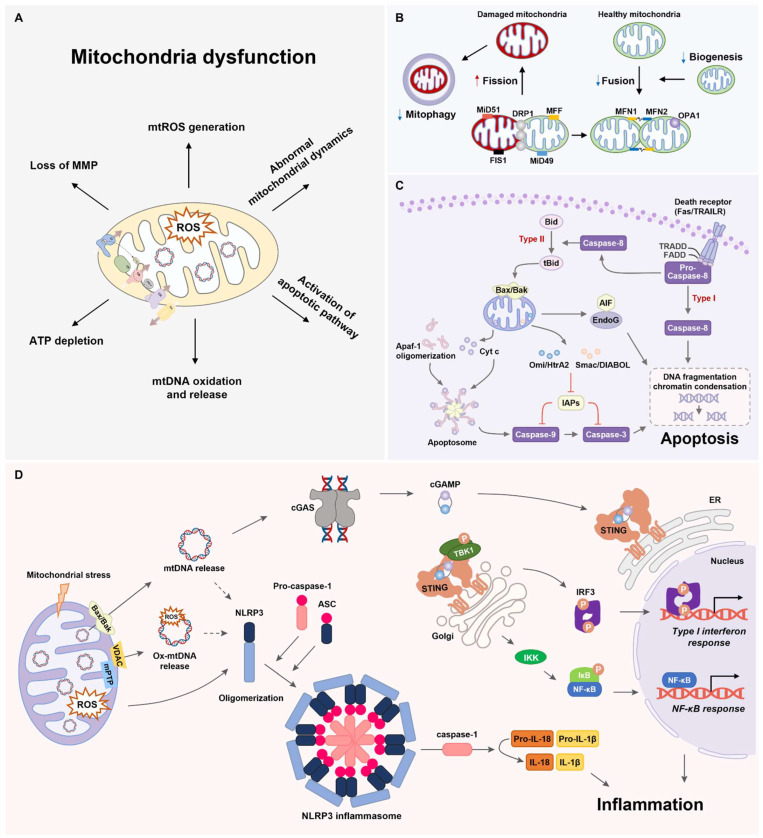
Summary of mechanisms of mitochondrial dysfunction. (**A**) Representative pathways of mitochondrial dysfunction. (**B**) A simplified overview of the mitochondrial dynamics. (**C**) A generalized mechanism for the mitochondria apoptosis pathway. (**D**) Overview of mitochondria-driven inflammation via the cGAS−STING signaling pathway and NLRP3 inflammasome pathway.

Mitochondrial fusion involves the physical merging of two mitochondria to produce distinctly healthy mitochondria. In mammalian cells, mitochondrial fusion involves mitofusin (MFN) 1, MFN2, and optic atrophy 1 (OPA1) located in the OMM and IMM [28]. MFNs, dynamin-like GTPases anchored in the OMM, facilitate OMM fusion via GTP hydrolysis. MFN1 and MFN2 share substantial similarities and compensate for each other [6,28]. MFN2, which is present in the endoplasmic reticulum (ER), aids in ER−mitochondrial tethering and influences mitochondrial fission [29]. OPA1, a GTPase in the IMM, governs IMM fusion, with alternative splicing producing the long (L−OPA1) and short (S−OPA1) forms. Cardiolipin, the specific lipid component of mitochondria, is crucial for IMM fusion. MFN synthesis is transcriptionally and post-transcriptionally regulated, and its degradation is controlled by ubiquitination and phosphorylation [30]. OPA1 undergoes post-transcriptional and post-translational regulation, and proteolytic processing plays a key role. Deficiencies in the fusion proteins led to mitochondrial fragmentation [31] (Figure 2).

### 3.2. Mitochondrial Fission

The initiation of mitochondrial fission involves mtDNA replication in the matrix, which marks the ER recruitment site [32]. OMM constriction begins at the mitochondrial−ER contact sites before dynamin-related protein 1 (DRP1) oligomerization [33]. Mitochondrial fission is tightly regulated by the recruitment of various mitochondria−bound proteins, such as mitochondrial fission 1 (FIS1), mitochondrial fission factor (MFF), mitochondrial dynamics 49 (MiD49), MiD51, and DRP1 [33]. DRP1 is a major fission protein that translocates from the cytosol to the OMM, where it oligomerizes and divides the mitochondrion into two separate organelles to remove damaged mitochondria. FIS1, which is implicated in the lysosomal marking of fission sites, is involved in inter-organelle communication during this process [34]. GTP hydrolysis induces conformational changes that enhance the ER-mediated constriction [35].

Although FIS1 is not essential for DRP1 recruitment or the induction of fission, FIS1 overexpression induces mitochondrial fragmentation without DRP1 [34]. Recent findings reveal the role of FIS1 in promoting mitochondrial fragmentation by activating fission; inhibiting fusion; and blocking the GTPase activity of MFN1, MFN2, and OPA1 [28,31,34]. This underscores the close connection between the fission and fusion processes and suggests potential mechanisms of mutual inactivation.

OMM constriction is well-documented; however, the mechanism of IMM division remains unclear. A recent study proposed Ca^2+^−dependent IMM constriction at mitochondrial−ER contact sites, suggesting a role for S−OPA1 in untethering the OMM from the IMM during fission [36]. However, further studies are required to comprehensively confirm and understand IMM fission.

In cells lacking DRP1, mitochondria exhibit a hypertubular configuration with a highly connected network [7]. Notably, DRP1 and its receptors localize to peroxisomal membranes, affecting processes beyond mitochondrial fission. DRP1 is regulated by phosphorylation, ubiquitination, and SUMOylation [37] (Figure 2). Factors such as exercise and circadian rhythms influence DRP1 phosphorylation; however, the underlying mechanisms remain unknown.

### 3.3. Mitophagy and Mitochondrial Biogenesis

Mitochondrial quantity and quality are coordinately regulated by two opposing processes, mitophagy and mitochondrial biogenesis, which respond to cellular energy demands and various intracellular or environmental signals [38,39]. Mitochondrial biogenesis involves increasing mitochondrial mass and number and the synthesis of new mitochondrial components, including mtDNA, proteins, and membranes, derived from existing mitochondria [38,39]. Peroxisome proliferator-activated receptor gamma coactivator−1α (PGC−1α) plays a critical role in regulating mitochondrial biogenesis by interacting with transcription factors nuclear respiratory factor 1 and 2 (Nrf1/Nrf2), which in turn, regulate mitochondrial transcription factor A (Tfam) expression, leading to mtDNA replication and transcription [13,39]. PGC−1α is activated through phosphorylation by AMP-activated protein kinase (AMPK) and deacetylation by Sirtuin 1 (SIRT1) [39].

Mitophagy selectively eliminates impaired and dysfunctional mitochondria to prevent further cellular damage by inhibiting the release of pro-apoptotic proteins [38]. The PTEN-induced kinase 1 (PINK1)/E3 ubiquitin ligase (PARKIN)-mediated pathway is the predominant and well-characterized mechanism of mitophagy [18,38]. Under normal conditions, PINK1 is imported into the inner mitochondrial membrane (IMM) and degraded by mitochondrial proteases and the proteasome. However, in damaged mitochondria, in which the IMM is depolarized, PINK1 accumulates on the outer mitochondrial membrane (OMM) in association with the translocase of the outer membrane (TOM) and the translocase of the inner membrane (TIM23) complexes, leading to PINK1 kinase activation [40]. Activated PINK1 then recruits PARKIN to the OMM and phosphorylates mitochondrial ubiquitin and the ubiquitin-like domain of PARKIN [18,40]. PARKIN-mediated ubiquitination targets several OMM proteins, including MFN1, MFN2, and the voltage-dependent anion channel (VDAC). Subsequently, the ubiquitinated mitochondria are recognized by the autophagy receptor P62/SQSTM1 and combined with LC3-mediated autophagosomes, forming mitophagosomes that undergo lysosomal degradation to remove damaged mitochondria [18,38].

### 3.4. Mitochondrial Dynamics and ROS

Recently, the interplay between ROS and mitochondrial dynamics was uncovered, indicating a correlation between cellular redox balance and the control of mitochondrial shape [41]. In the absence of an effective antioxidant defense system, elevated ROS levels tend to induce mitochondrial fragmentation, swelling, or shortening, whereas a reduction in ROS levels promotes mitochondrial elongation [4,23]. For example, in human umbilical vein endothelial cells (HUVECs), exogenous H_2_O_2_ at varying concentrations triggers dose-dependent mitochondrial fragmentation and influences the expression of genes related to fusion and fission [42]. Similarly, in C2C12 myocytes, H_2_O_2_ leads to mitochondrial depolarization and enhanced fragmentation by upregulating DRP1 activity [43]. Conversely, decreased ROS levels in fibroblasts results in MFN2−dependent mitochondrial elongation. Redox control of fission and fusion proteins by ROS involves various post-translational modifications, such as phosphorylation, ubiquitination, and SUMOylation, as well as the S−glutathionylation and S−nitrosylation of cysteine residues. Furthermore, ROS exerts transcriptional effects by stimulating the expression of factors implicated in redox regulation and mitochondrial dynamics [37]. For instance, PGC−1α, which is sensitive to redox changes, is associated with the regulation of MFN2. AMPK also plays a crucial role in the crosstalk between mitochondrial dynamics and ROS [44]. Upon activation, AMPK phosphorylates MFF and DRP1, which is essential for mitochondrial fission.

### 3.5. Mitochondrial Inheritance

Mitochondria, which are vital for cell division, exhibit dynamic changes in their organization during the cell cycle. In mammalian cells, prior to mitosis and cytokinesis, mitochondrial network fragments allow for the stochastic distribution of organelles to daughter cells [45]. Interestingly, yeast cells demonstrate the orderly inheritance of mitochondria through cytoskeleton-dependent transport, and mutants lacking certain mitochondrial division proteins surprisingly maintain their viability without significant growth defects.

In yeast, the disruption of mitochondrial fusion leads to rapid loss of the mitochondrial genome and respiratory defects. This is attributed to the generation of multiple small organelles and lack of mtDNA, resulting in the inheritance of progeny with significantly depleted mtDNA [46]. Consequently, mitochondrial genomes are lost from the population after several generations.

In mammalian cells, the disruption of mitochondrial fusion leads to mitochondrial heterogeneity and dysfunction. This dysfunction may be linked to the loss of nucleoids in individual mitochondria. MFN or DRP1 knockout in mice results in prenatal death, underscoring the crucial role of mitochondrial dynamics in mammalian development [7,47]. The fusion process plays a fundamental role in maintaining the mitochondrial population with a full complement of nuclear- and mitochondrion−encoded gene products [48]. While mitochondrial fission generates organelles lacking nucleoids, fusion ensures the timely replenishment of the mitochondrial genome and gene products, thereby preventing functionality loss [49].

### 3.6. Mitochondrial Distribution and Morphology

Cells with defective mitochondrial division exhibit interconnected net-like mitochondria that accumulate in specific areas, leaving other parts of the cell devoid of mitochondria [50]. Proper mitochondrial distribution, which is crucial for large cells, such as neurons, relies on division to create transportable units. DRP1 and OPA1 play essential roles in establishing the appropriate mitochondrial content in dendrites [51], which is crucial for maintaining dendritic spines and synapses that are sites of high energy demand. DRP1-knockout mice show severe developmental abnormalities, particularly in the forebrain and cerebellum [47,52]. Neurons with DRP1 deficiency accumulate mitochondria in the cell body, hindering their proper distribution. Mice with conditional MFN2-knockout alleles exhibit Purkinje neuron degeneration due to fragmented mitochondria and mitochondrial distribution failures, suggesting an impact of fusion on distribution [53].

In muscle cells, fused mitochondrial networks function as electrically united systems that transmit MMP, aiding energy dissipation [54]. This is vital in large muscle cells, in which mitochondrial filaments connect regions with different oxygen levels, facilitating energy dissipation and ATP production during muscle contraction [54,55]. Mitochondrial fusion increases under stress conditions involving ATP production and during the G1 to S transition, consistent with its role in energy dissipation [55]. The coordinated activities of the mitochondrial fusion and fission machinery adapt to the mitochondrial compartment to meet specific cellular requirements.

## 4. Mitochondrial Apoptosis Pathway

Apoptosis is a well-coordinated cellular defense system that closely regulates processes in which ligand binding to cell death-related receptors or cytotoxic insults results in the activation of diverse proteases and the stimulation of other hydrolytic enzymes, leading to DNA fragmentation and proteolysis [56]. Programmed cell death can occur through intrinsic or extrinsic pathways, relying on signals leading to apoptosis. Extrinsic signals (such as glucocorticoids, cytokines, Fas ligand, and tumor necrosis factor [TNF] −α) bind to receptors and activate cellular signaling leading to caspase−8 activation [57]. The Fas ligand and TNF pathways induce both cell survival and apoptosis depending on the intracellular signaling pathways [58].

In the intrinsic pathway, apoptotic markers (such as Bax and Bak) translocate to mitochondria and permeabilize the mitochondrial membrane [59,60]. These translocations induce the release of several intermembrane proteins (such as Smac/DIABOL, apoptosis-inducing factor [AIF]/Endo G, and cytochrome c [Cyt c]) into the cytosol [61]. Cyt c, released into the cytosol, stimulates the apoptosome complex and activates caspase−9.

The apoptosome consists of apoptotic protease activating factor 1 (APAF-1), Cyt c, and pro-caspase−9. Apoptosome-activated caspase-9 supports the cleavage of caspase−3 and stimulates its proteolytic activation [62]. This results in the manifestation of the following two critical apoptotic characteristics: the exposure of phosphatidylserine on the external surface of the plasma membrane and the fragmentation and degradation of DNA. Recent research indicates that caspase−3 may additionally play a role in amplifying the initial cell death signal by facilitating the release of Cyt c from mitochondria [56]. Smac/DIABLO promotes apoptosis by binding to and counteracting members of the inhibitor of the apoptosis protein complex [63]. In contrast, AIF and EndoG translocate from the cytosol to the nuclear compartment, initiating DNA fragmentation [64] (Figure 2). The mitochondrial intermembrane space protein-release mechanisms remain controversial, with two proposed mechanisms depending on the nature of the apoptotic stimulus. The first involves a mitochondrial permeability transition pore (mPTP) opening in the inner membrane that allows the passage of water and molecules [20]. The key components include the adenine nucleotide transporter and the voltage-dependent anion channel. Activation leads to the equilibration of ions, causing a loss of MMP and matrix swelling. Subsequent outer membrane breakage results in the release of pro-apoptotic factors into the cytosol [65].

The second mechanism involves the Bcl-2 family subunits acting directly on the outer mitochondrial membrane, where the oligomerization of pro-apoptotic Bax and Bak is crucial for membrane permeabilization [59,66]. Translocation of Bax/Bak to mitochondria induces ROS generation and cardiolipin (CL) oxidation. Cells lacking both Bax and Bak are resistant to apoptotic stimuli, including oxidants, in contrast to cells lacking only one of these proteins [60]. Regardless of the mechanism, permeabilization of the mitochondrial outer membrane is considered an irreversible step in programmed cell death due to the release of caspase activators, such as Cyt c (Figure 2).

Mitochondria play an important role in the intrinsic and extrinsic apoptosis pathways [57]. The intrinsic pathway is mitochondria-dependent, but cells in the extrinsic pathway are categorized as type I or II based on mitochondrial involvement [67]. Type I cells undergo apoptosis without significant mitochondrial participation, which is crucial for developmental tissue remodeling [68]. Type II cells contain mitochondria as a secondary loop during apoptosis [57]. In certain death receptor-mediated systems, extrinsic stimuli activate caspase-8, initiating mitochondria-dependent signaling. This process leads to the cleavage of the pro-apoptotic Bcl-2 family protein Bid into t-Bid, which translocates to the mitochondria and triggers mitochondrial events during apoptosis [59].

## 5. Mitochondria-Associated Inflammation

Mitochondria regulate the innate immune system through the following two primary pathways: the cyclic GMP−AMP synthase (cGAS)−stimulator of interferon genes (STING) signaling and the NLR pyrin domain containing 3(NLRP3) inflammasome signaling [69,70]. Mitochondrial damage may induce the efflux of mtDNA into the cytosol via BAX/BAK macropores, mPTP opening, and VDAC oligomerization [16,69]. The cGAS−STING pathway has emerged as a crucial mechanism linking the detection of cytosolic mtDNA to the initiation of inflammatory responses [56,70]. cGAS acts as a cytosolic double-stranded DNA sensor that recognizes cytosolic mtDNA and catalyzes the production of the second messenger molecule 2′3′ cyclic GMP−AMP (cGAMP) [69,70]. Upon the binding of cGAMP to STING dimers, STING undergoes oligomerization and translocates from the ER to the cis-Golgi network. Subsequently, STING migrates to the trans-Golgi network where it clusters through palmitoylation-dependent accumulation of cholesterol [71]. The clustering of STING facilitates the recruitment and activation of TANK-binding kinase 1 (TBK1), resulting in STING phosphorylation [56,69,71]. Active TBK1 then phosphorylates the transcription factor interferon regulatory factor 3 (IRF3), promoting IRF3 dimerization and translocation to the nucleus to initiate the type I interferon (IFN) response [16,56,69,70]. Moreover, STING activates the NF-κB signaling pathway by phosphorylating the IκB/IκB kinase (IKK) complex, leading to the upregulation of proinflammatory cytokines, such as IFN−β1, interleukin-6 (IL−6), and TNF [56,69].

The NLRP3 inflammasome signaling is activated by both mtDNA and mitochondrial ROS released from stressed mitochondria [56,69]. This multiprotein complex comprises the cytosolic sensor NLRP3, the adapter protein apoptosis-associated speck-like protein containing a CARD (ASC), and the effector pro-caspase-1, leading to caspase-1-mediated proteolytic maturation and the secretion of IL−1β and IL−18 [69,72,73]. Oxidized mtDNA (Ox−mtDNA) is generated when mtDNA is exposed to mitochondrial ROS during mitochondrial dysfunction, and it is released into the cytosol through mPTP- and VDAC-dependent channels. The accumulation of Ox−mtDNA in the cytosol binds to NLRP3, triggering the assembly and activation of the NLRP3 inflammasome, along with STING Ser365 phosphorylation [72]. However, the mechanism by which mtROS and mtDNA activate the NLRP3 inflammasome remains unclear due to the absence of DNA recognition or binding domains within the NLRP3 receptor. Optimal NLRP3 inflammasome activation involves physical interactions between NLRP3 and cardiolipin, a mitochondrial phospholipid, as well as thioredoxin-interacting protein (TXNIP), a pro-oxidant protein that translocates from the nucleus to the mitochondria under oxidative and endoplasmic reticulum (ER) stress [12,56]. Interestingly, STING also activates the NLRP3 inflammasome in response to cytosolic DNA stimulation by promoting NLRP3 localization in the ER and facilitating NLRP3 inflammasome formation. STING interacts with NLRP3 to inhibit K48- and K63-linked polyubiquitination, thereby promoting NLRP3 inflammasome activation [74]. Thus, various mitochondrial components and products released during mitochondrial dysfunction promote inflammatory responses through the activation of cGAS−STING signaling and NLRP3 inflammasome signaling. Although recent studies have focused on the relationship between the cGAS−STING signaling axis and the NLRP3 inflammasome due to their similarities in responding to cellular stress and their downstream effects, their biological relevance and physical interaction have yet to be confirmed. Significant questions remain, including whether the NLRP3 inflammasome can activate the cGAS−STING signaling pathway, which signaling pathway is predominantly activated by specific cellular stress conditions, and which signaling pathway contributes more to the progression of inflammation-related diseases.

## 6. Mitochondria Dysfunction and Oxidative Stress in the Pathogenesis of Neurodegenerative Diseases

AD and PD are the most common age-related neurodegenerative diseases that affect millions of people worldwide [10,11]. Although the primary causes of these diseases remain unknown, emerging evidence indicates that mitochondrial dysfunction and oxidative stress are involved in the development and progression of neurodegenerative diseases [10].

### 6.1. Alzheimer’s Disease

AD is a major neurodegenerative disease that is strongly associated with memory loss, cognitive decline, and dementia in the elderly. AD is characterized by the extracellular deposition of amyloid β (Aβ) plaques and the formation of neurofibrillary tangles that consist of abnormally accumulated microtubule-associated protein tau in the brain, leading to neuronal toxicity in the central nervous system [11,13].

#### 6.1.1. Oxidative Stress

Aβ treatment causes ROS accumulation and Bcl-2-interacting protein 3 (BNIP3)-mediated mitochondrial dysfunction, which results in the death of rat primary cortical neurons. Aβ-induced neuronal death is attenuated by antioxidant vitamins and BNIP3 knockdown [75,76]. Significant increases in the lipid peroxidation product 4-hydroxynonenal, the protein oxidation product protein carbonyl, and the protein nitration product 3-nitrotyrosine have been observed in the brains of individuals with AD and mild cognitive impairment (MCI). The oxidative modification of proteins involved in key cellular processes results in protein dysfunction or loss of function [76]. In contrast, a reduction in antioxidant glutathione levels has also been found in brain regions of patients with AD and MCI [77]. These studies have confirmed that Aβ-induced oxidative stress leads to increased protein oxidation and impairment of the antioxidant system, contributing to the pathogenesis of AD [76,77]. In addition, increased ROS production causes tau hyperphosphorylation through glycogen synthase kinase 3β activation, and this may be related to learning and memory impairment [11,78]. Thus, increased oxidative stress has been implicated in the development and progression of AD.

#### 6.1.2. Alterations in Mitochondrial Dynamics

Abnormal mitochondrial dynamics cause excessive mitochondrial fragmentation and dysfunction, leading to a reduction in OXPHOS, ATP production, and MMP and an elevation in ROS generation [11,13]. Importantly, significantly decreased levels of the fusion factors OPA1, MFN1, and MFN2 and significantly decreased levels of the fission factors DRP1 and FIS1 have been found in the brains of individuals with AD [79]. Aβ treatment induces the translocation of DRP1 to mitochondria and its phosphorylation at Ser616 via the activated AKT pathway, and this stimulates mitochondrial fission. Moreover, Aβ−mediated AKT activation inhibits autophagy through the mammalian target of rapamycin (mTOR) pathway and promotes excessive mitochondrial fission, resulting in ROS-mediated neuronal death [80]. NO production in response to Aβ protein triggers mitochondrial fission, synaptic loss, and neuronal injury via S-nitrosylation of DRP1 [81]. Abnormal interactions between Aβ and DRP1 and between Aβ and hyperphosphorylated tau increase mitochondrial fragmentation, neuronal and synaptic damage, and cognitive decline in patients with AD and mouse models of AD [79,82]. Notably, the impairment of mitophagy has been observed in the hippocampus of AD patients and in iPSC−derived neurons from AD patients due to reduced levels of activated mitophagy proteins, such as PINK1, BNIP3, TBK1, and ULK1. Mitophagy stimulation by urolithin A treatment diminishes insoluble Aβ1–42 levels, extracellular Aβ plaque formation, and NLRP3 inflammasome-mediated neuroinflammation in Aβ precursor protein (APP)/PS1 transgenic mice. Moreover, urolithin A-induced mitophagy reduces mitochondrial ROS and tau hyperphosphorylation and reverses memory loss in transgenic tau nematodes and 3xTgAD mice [83]. Impaired mitochondrial fusion as a result of MFN2 ablation increases the number of swollen and fragmented mitochondria with broken cristae; mitochondrial dysfunction; the oxidative stress response; and eventually, neuroinflammation-mediated cell death. After MFN2 knockout, the hippocampus and cortex exhibit severe neurodegeneration with neuron loss and a reduction in hippocampal and cortical size, all of which are pathological features of AD [84]. A recent study reported enhanced tau acetylation at K274 and K281 in the brains of patients with AD and in mouse models of AD. The overexpression of K274/K281 mutant tau disrupts mitochondrial dynamics by decreasing the expression levels of the fusion proteins MFN1, MFN2 and OPA1 and by inhibiting biogenesis through the PGC−1α/Nrf1/Tfam signaling pathway, which confirms that acetylated tau exacerbates severe cognitive deficits with neuronal loss and dendritic plasticity damage [85]. The 5xFAD transgenic mouse model of AD exhibits an impaired expression of the biogenesis proteins Tfam and SIRT3, the fusion protein MFN2, and the fission proteins MTP18 and DRP1 in the hippocampus. Additionally, the PINK1/PARKIN−dependent mitophagy pathway, MICU1/MCU−mediated mitochondrial Ca^2+^ uptake, and maximal respiration are defective in 5xFAD mice as early as two months of age [86]. Therefore, mitochondrial dysfunction caused by an imbalance in mitochondrial fission and fusion plays an important role in the pathogenesis of AD.

#### 6.1.3. Mitochondria-Driven Inflammation

Various studies highlight the pivotal role of mitochondria-associated neuroinflammation in the onset and progression of AD [56,69,87,88]. It has been shown that the main proinflammatory cytokine IL−1β is overexpressed in the brains of patients with AD [87]. Both Aβ oligomers and tau aggregates trigger the assembly and activation of the NLRP3 inflammasome in the brain, leading to caspase-1 activation, excessive IL−1β production, and neuronal degeneration. Crossing APP/PS1 mice with NLRP3^–/–^ or caspase-1^–/–^ mice improves cognitive behavior, synaptic function, and Aβ clearance [87,89]. The pharmacological inhibition of the NLRP3 inflammasome using OLT1177, an NLRP3 inhibitor, and VX−765, a caspase−1 inhibitor, significantly reduces IL−1β levels, thereby preventing microglial activation and memory deficits in AD mouse models [87,89]. Additionally, the activation of the cGAS−STING pathway is evident in the brains of aged Tau P301S transgenic mice, 5xFAD transgenic mice, and human AD patients [88,90]. Pathogenic tau activates cGAS−IFN signaling through cytosolic mtDNA, impairing cognitive resilience by disrupting the neuronal transcriptional network regulated by myocyte enhancer factor 2c (MEF2C). The genetic deletion of cGAS and treatment with cGAS inhibitors enhance the expression of MEF2C target genes, restoring synaptic integrity, plasticity, and memory in Tau P301S transgenic mice [90]. Furthermore, the cGAS−STING pathway contributes to AD pathogenesis, as evidenced by cognitive assessments, Aβ pathology, and neuroinflammatory responses in cGAS^–/–^; 5xFAD mice. The cGAS inhibitor RU.521 and STING inhibitor H−151 demonstrate potent neuroprotective effects against oligomeric Aβ42−induced neuronal toxicity [88].

### 6.2. Parkinson’s Disease

PD is a neurodegenerative disease characterized by motor impairment and non-motor symptoms [91]. The pathological hallmarks are the selective death of dopaminergic neurons in the substantia nigra pars compacta, leading to abnormal dopamine metabolism and the appearance of Lewy bodies and Lewy neurites composed of aggregated α-synuclein [92,93]. Although the precise factors leading to dopaminergic neuronal cell death remain unclear, several causes are responsible for the progression of PD, including the accumulation of aggregated protein, disruption of the ubiquitin-proteasome pathway, neuroinflammation, and mitochondrial dysfunction [9,94]. Mitochondrial dysfunction is a major factor affecting the initial stages of PD pathogenesis. In particular, the inhibition of mitochondrial complex I activity is accompanied by oxidative stress and the breakdown of mitochondrial Ca2+ homeostasis in dopaminergic neurons of patients with PD [9,95].

#### 6.2.1. Oxidative Stress

Environmental neurotoxins include 1−methyl−4-phenyl−1,2,3,6−tetrahydropyridine (MPTP), 6−hydroxydopamine (6−OHDA), and rotenone. MPTP is a representative neurotoxin used to generate an ideal animal model of PD [96]. MPTP is metabolized to the active form, 1−methyl−4−phenylpyridinium (MPP^+^), in glial cells, and MPP^+^ is then released into dopaminergic neurons via the dopamine transporter. Released MPP^+^ suppresses the activity of mitochondrial complex Ⅰ and subsequently enhances excess ROS generation, resulting in the death of the dopaminergic neurons, similar to the parkinsonian phenotype [97].

#### 6.2.2. Impaired Mitochondrial Dynamics and Function

Several studies have reported that genetic factors such as *PARKIN*, *PINK1*, *SNCA*, *F*−*box protein only 7*/PARK15 *(FBXO7)*, *DJ*−*1*, and *Leucine-rich repeat kinase 2 (LRRK2)* contribute to mitochondrial impairment in patients with PD [98]. PINK1 and PARKIN are well-known representative factors of autosomal recessive and early-onset PD [99]. The interaction between PINK1 and PARKIN is considered the first step in protecting against mitochondrial dysfunction [100]. In the pathology of PD, the deletion of PINK1 and PARKIN fails to maintain mitochondrial quality and accelerates disease [101]. A recent study sheds new light on how PINK1 regulates mitophagy in response to mitochondrial stress. The N−terminal−C-terminal extension (NT−CTE) module of PINK1 interacts with the Tom20 subunit of the TOM complex, leading to the formation of a supercomplex with the TOM and TIM23 complexes. This PINK1−TOM−TIM23 supercomplex is crucial for the clearance of dysfunctional mitochondria. Mutations in PD-associated PINK1 (I111S, C125G, Q126P, A536S, and R543G) within the NT–CTE region disrupt the assembly of the PINK1 supercomplex, impairing downstream mitophagy. Therefore, the PINK1–TOM–TIM23 supercomplex plays a critical role in controlling mitochondrial quality in PD pathology [40]. The A53T mutation in α-synuclein, encoded by SNCA, leads to a reduction in mitochondrial respiration and MMP, as well as abnormal mitochondrial morphology. iPSC-derived dopaminergic neurons from PD patients carrying the A53T SNCA mutation exhibit reductions in basal and maximal respiration and ATP production, despite no changes in the total mitochondrial mass. The mitochondria in these neurons appear circular, unbranched, and more rounded, resembling a donut shape [102]. Moreover, this mutation can cause changes in the levels of several mitochondrial proteins, including DRP1, MFN, OPA1, and PGC-1α. Mutations in FBXO7 are strongly associated with autosomal recessive early-onset PD [103]. Recent findings have demonstrated that mice with neuron-specific conditional FBXO7 knockout display small, round, and fragmented mitochondria and a reduced mitochondrial area and are tyrosine hydroxylase-positive in dopaminergic neurons [104]. LRRK mutations are observed in late-onset autosomal-dominant forms of PD [105]. In brain tissue from patients with PD and rat models of idiopathic PD, wild-type LRRK2 kinase activity is markedly amplified through an oxidative mechanism, leading to defects in mitochondrial protein (complex Ⅰ subunit Ndufs3) import [106]. In addition, LRRK2 deletion, inhibition, and mutation lead to mitochondrial Ca^2+^ efflux through Na^+^/Ca^2+^/Li^+^ exchange, which is linked to changes in the mPTP and dopaminergic neuronal cell death [107]. The loss of coiled−coil−helix−coiled−coil−helix domain−containing 2 (CHCHD2) stability has also been observed in autosomal-dominant PD patients. The CHCHD2 gene is well-known as the first mitochondrial gene related to PD progression [108,109,110]. Additionally, p32 plays a crucial role in regulating CHCHD2 stability in PD pathology. In PD drosophila transgenic models, both p32 knockdown and a p32 inhibitor ameliorate PD phenotypes by reducing CHCHD2 mutations and their downstream effects. They restore muscle mitochondrial morphology and dopaminergic neuronal numbers compared to PD models. Furthermore, treatment with a specific p32 inhibitor (p32−I) enhances dopaminergic neuron differentiation, increases neurons expressing tyrosine hydroxylase, extends neurite length, and reduces α-synuclein levels in hCHCHD2 Arg145Gln neural progenitor cells [111].

#### 6.2.3. Mitochondria-Activated Inflammatory Pathway

Chronic neuroinflammation has been reported in the nigrostriatal system of living PD patients and postmortem PD brains [92,112,113]. Elevated levels of NLRP3, ASC, and cleaved caspase-1 proteins are observed in the substantia nigra of the brains of PD patients and in multiple PD models, including 6-OHDA−administered mice, dopaminergic neuron-specific Tfam^–/–^ (MitoPark) mice, and α-synuclein pre-formed fibril (PFF)−injected mice. These elevations are accompanied by mitochondrial dysfunction, oxidative stress, and α-synuclein pathology [112,114]. Importantly, treatment with fibrillar α-synuclein robustly activates the NLRP3 inflammasome, resulting in the extracellular release of ASC, cleaved caspase−1, and cleaved IL−1β in primary microglia [112]. In hiPSC−derived microglia, oligomeric α-synuclein activates the NLRP3 inflammasome through toll-like receptor 2 (TLR2) stimulation and causes mitochondrial damage, including mtROS overproduction, mtDNA release, and MMP reduction. The engraftment of hiMG with α-synuclein oligomers into the brains of humanized mice induces caspase−1 activation and caspase−3−mediated neuronal cell death [115]. These findings highlight that microglial NLRP3 inflammasome activation can be a sustained source of neuroinflammation, contributing to progressive dopaminergic neuropathology in PD [112,113,114,115]. Collectively, the close relationship between mitochondrial damage and ROS significantly contributes to the pathogenesis of both AD and PD. Therefore, targeting mitochondrial dysfunction and ROS is a promising strategy for drug development aimed at treating AD and PD.

## 7. Mitochondrial Dysfunction and ROS as Therapeutic Targets for Neurodegenerative Diseases

There are currently no effective treatments for AD or PD. Symptomatic treatments are available, but their effects are limited, and they can only slow down disease progression [9,10,11]. Thus, there is a clear need to develop novel targets for the treatment of both AD and PD. Numerous studies have demonstrated that blocking mitochondrial dysfunction and oxidative stress, as well as improving mitochondrial dynamic homeostasis, may be valuable therapeutic strategies for new AD and PD drugs (Table 1).

### 7.1. Mitochondrial Fusion and Biogenesis Enhancers

SS−31 (also known as Elamipretide, Bendavia, and MTP-131) is a mitochondria-targeting tetrapeptide that can protect mitochondrial function through its mitochondria-targeted antioxidant activity in both AD and PD models [116,117,118]. The MPP^+^ stimulation of dopaminergic neurons leads to the inhibition of oxygen consumption and ATP production and the induction of mitochondrial swelling and cell death, which are rescued by SS−31 treatment [116]. SS−31 prevents Aβ neurotoxicity by increasing the expression levels of antioxidant enzymes Prxs and decreasing the expression levels of mitochondrial fission proteins DRP1 and FIS1 in N2a neuroblastoma cells [117]. Furthermore, SS−31 treatment increases the expression levels of mitochondrial biogenesis-related proteins PGC−1α, Nrf1/2, and Tfam, as well as the mitochondrial fusion proteins MFN1and MFN2. It suppresses mitochondrial dysfunction, particularly the loss of Cyt c activity and MMP and ATP production in APP transgenic mice [118]. Ultimately, the Aβ production, synaptic loss, and cognitive impairments observed in APP/PS1 transgenic mice are reversed by long-term treatment with SS-31 [119].

BGP−15 is a small hydroxylamine compound that has cytoprotective effects in animal models of several oxidative stress-related diseases through the inhibition of mitochondrial ROS production and the c-Jun N-terminal kinases (JNK)/AIF/caspase pathway and the activation of the AKT/mTOR pathway and extracellular signal-regulated kinase phosphorylation [128]. In particular, BGP−15 has neuroprotective effects that promote mitochondrial fusion by increasing MFN1, MFN2, and OPA1 levels and mitochondrial biogenesis by activating the PGC−1α/Nrf1/Tfam pathway. BGP−15 treatment rescues acetylated tau−mediated neurotoxicity and cognitive deficits by attenuating mitochondrial dysfunction, neuronal loss, and dendritic complexity damage in an AD model [85].

### 7.2. Mitochondrial Fission Inhibitors

Mitochondrial division inhibitor 1 (Mdivi−1) is a cell-permeable selective DRP1 inhibitor that inhibits DRP1−dependent mitochondrial fission and induces neuroprotection in AD and PD models [80,120]. Mitochondrial fission inhibition by Mdivi−1 mitigates ROS generation and loss of MMP and ATP production in Aβ−treated SK−NK−SH neuroblastoma cells. Mdivi-1 treatment also attenuates Aβ−induced mitochondrial apoptosis by decreasing activation of the Cyt c/caspase−9/caspase−3 pathway [80]. Furthermore, Mdivi−1 preserves motor function and dopamine levels and protects against nigrostriatal degeneration in A53T α-synuclein−induced neurotoxicity. Mechanistically, human A53T α-synuclein overexpression in a rat model induces the aggregation and toxic phosphorylation of α-synuclein, ROS−mediated lipid peroxidation, mitochondrial fragmentation, and mitochondrial dysfunction, which are prevented by Mdivi-1 treatment [120].

P110 is a seven-amino acid peptide that inhibits DRP1 enzyme activity and blocks the DRP1-FIS1 interaction, whereas it has no effect on the interaction between DRP1 and other mitochondrial adapters [121,122,123]. The inhibition of DRP1 hyperactivation by P110 reduces mitochondrial fragmentation and damage by inhibiting mitochondrial ROS production and improving MMP and mitochondrial integrity in MPP^+^−treated SH−SY5Y neuronal cells and primary rat dopaminergic neurons. P110 mitigates neuronal cell death by suppressing the Bax/Cyt c−induced mitochondrial apoptotic pathway and LC3−mediated autophagy in a PD model [121]. Additionally, P110 exerts neuroprotective effects in MPTP-induced PD mice by blocking dopaminergic neuron apoptosis through the inhibition of DRP1-dependent p53 mitochondrial translocation [122]. Meanwhile, P110 treatment alone decreases the expression levels of APP and β-site APP cleaving enzyme 1 and increases the expression levels of a disintegrin and metalloproteinase domain-containing protein 10 and klotho, which act as neuroprotective proteins in SH−SY5Y neurons [123]. Thus, P110 may be useful in attenuating Aβ formation and enhancing neuroprotection in AD models.

### 7.3. Antioxidants

Coenzyme Q_10_ (CoQ_10_), also known as ubiquinone, is an essential cofactor in the ETC and the most widely used antioxidant tool in in vitro and in vivo experiments. CoQ_10_ is a vitamin-like fat-soluble molecule known as a strong antioxidant, anti-inflammatory, and neuroprotective agent. Several studies have established that CoQ_10_ treatment supports mitochondrial function by enhancing ETC, MMP, and ATP synthesis and maintaining the cellular redox balance via antioxidant enzyme activation and a reduction in NOS expression levels to suppress ROS/RNS generation [10,11,124,125]. Aβ-injected adult rats show impaired hippocampal long-term potentiation and increased total oxidant levels, which are reversed by improving antioxidant activity through the oral administration of CoQ_10_ [125]. In addition, DRP1/FIS1-mediated mitochondrial fragmentation induced by rotenone, an inducer of PD, is attenuated in HT22 neuronal cells treated with water-soluble CoQ_10_ [124].

Mitoquinone mesylate (MitoQ) is a selective mitochondria-targeted antioxidant that eliminates mitochondrial ROS and enhances mitochondrial function [11]. MitoQ consists of a lipophilic triphenylphosphonium cation linked to the ubiquinone antioxidant moiety of CoQ_10_. It can enter the mitochondrial matrix and accumulate at levels several hundred times greater than those of natural antioxidants [117,127]. MitoQ treatment of Aβ-stimulated N2a cells reduces lipid peroxidation and H2O2 generation, restores the mitochondrial fragmentation and abnormal expression of mitochondrial dynamics proteins, and subsequently improves neurite outgrowth and cell viability [117]. Solubilized human Aβ1–42 causes decreases in the protein levels of the synaptic markers synaptophysin and postsynaptic density protein 95 and the mitochondrial antioxidant SOD2 in the entorhinal cortex, and this is prevented by co-treatment with MitoQ [126]. Of note, MitoQ treatment particularly enhances MFN2-dependent mitochondrial fusion by activating PGC-1α and stabilizing mitochondrial morphology and function in 6−OHDA−treated SN4741 dopaminergic cells. Moreover, the administration of MitoQ to mice with 6-OHDA-induced PD significantly rescues the loss of dopaminergic neurons in the substantia nigra compacta [127].

### 7.4. Mitochondria-Driven Inflammation Inhibitors

MCC950 is a small-molecule NLRP3 inhibitor that directly interacts with the ATP-hydrolysis motif within the NACHT domain, blocking its ATPase activity and NLRP3 inflammasome assembly. It is the most potent and widely used NLRP3 inhibitor in research [73,114]. MCC950 abolishes the α-synuclein-induced release of cleaved caspase-1, cleaved IL-1β, and ASC, as well as ASC oligomerization in primary mouse microglia. Oral treatment with MCC950 in multiple mouse PD models blocks caspase-1 activation and effectively protects against motor deficits, nigrostriatal dopaminergic degeneration, and the accumulation of α-synuclein aggregates [112].

OLT1177 (Dapansutrile), a β-sulfonyl nitrile synthetic compound, is an orally active and selective NLRP3 inhibitor. OLT1177 inhibits the ATPase activity of NLRP3 and prevents NLRP3 inflammasome formation, subsequently suppressing downstream caspase−1 activation and maturation of IL-1β, IL-18, and IL-6. It has been shown to cross the blood–brain barrier and is safe in humans, with clinical trials for the treatment of inflammatory diseases such as acute gout flare [73,87,113]. In six-month-old APP/PS1 mice, the oral administration of OLT1177 for three months effectively rescues synaptic plasticity impairments in learning and memory based on the Morris water maze test. OLT1177 treatment reduces the number of cortical Aβ plaques and normalizes plasma AD metabolic markers, such as deaminated purines, glutathione turnover metabolites, and polyunsaturated fatty acids [87]. Furthermore, OLT1177 injection in MPTP−induced PD mice prevents loss of motor function and dopaminergic neuron degeneration and reduces levels of α-synuclein and proinflammatory IL−1β, IL−18, IL−6, and IL−17A in the brain [113].

VX−765 (Belnacasan) is a selective caspase−1 inhibitor that inhibits caspase−1 activity and IL−1β release [73]. It is an orally bioactive prodrug that rapidly metabolizes to VRT−043198, which is non-toxic and permeable to the blood–brain barrier [89]. VX−765 pre-symptomatic treatment in APPSw/Ind mutant J20 mice decreases active IL−1β levels and microglial inflammation, thereby preventing AD−related memory deficits, synaptic loss, and progressive Aβ accumulation [89].

TDI−8246 is a small molecule inhibitor of human cGAS, and TDI−6570 is a small molecule inhibitor of mouse cGAS. These are potent and specific inhibitors of cGAS with high gastrointestinal absorption and good brain permeability. TDI−6570 treatment in primary microglia diminishes HT-DNA-induced cGAS activation and cGAS−dependent IFN responses in a dose-dependent manner with sub-micromolar activity. Additionally, TDI−6570 administration reduces microglial IFN responses, enhances the neuronal MEF2C transcriptional network, and protects against synapse loss and cognitive deficits in mice with tauopathy [90].

H−151, a 3−acylamino indole derivative, is a selective and irreversible antagonist of STING that exerts its inhibitory action by covalently binding to STING at Cys91. H−151 strongly reduces IFN responses and TBK1 phosphorylation and blocks STING palmitoylation on Cys91 [70,71]. The administration of H-151 potently suppresses the activation of the cGAS−STING pathway, as indicated by reduced levels of p−TBK1, p−p65, and p−IRF3 proteins, and ameliorates Aβ pathology and neuroinflammation in 5xFAD mice [88].

## 8. Conclusions and Future Perspectives

In this review, we have examined recent research findings on mitochondrial function, specifically focusing on the ATP and ROS generation processes. We have also explored the roles of ROS-induced oxidative stress, abnormal mitochondrial dynamics, mitochondrial apoptosis, and impaired mitochondrial function in the pathogenesis of AD and PD. Furthermore, we have summarized various mitochondria-targeted compounds, including mitochondrial fusion and biogenesis enhancers, mitochondrial fission inhibitors, and antioxidants for intracellular and mitochondrial specificity, which hold potential as novel therapeutic drug candidates for AD and PD. While each of these mitochondria-targeted compounds has its distinct functions, enhancing each function ultimately leads to the overall improvement of mitochondrial function.

Recent studies have introduced high-throughput screening methods to develop novel mitochondria-targeted compounds for neurodegenerative diseases. For example, an assay using a mitochondrial-targeted fluorescence reporter, CAG>mtTagGFP2, can monitor alterations in mitochondrial dynamics by measuring mitochondrial content, length, and circularity [129]. Furthermore, the mitochondrial-targeted TMRM/ATP high-throughput screen for measuring MMP and ATP production can be used to identify multiple structural and functional classes of compounds that enhance mitochondrial function [130]. These advanced high-throughput screening platforms could be effectively used to search for hit compounds. Thus, developing high-throughput screening platforms is needed to evaluate the efficacy of newly developed mitochondria-targeted compounds.

Accumulating evidence from numerous recent studies indicates that mitochondrial dysfunction and mitochondrial ROS are closely associated with the inflammatory response. Inflammation driven by mitochondrial dysfunction has been shown to contribute to numerous human disorders, particularly AD and PD. Pharmacologic inhibitors of mitochondria-associated neuroinflammation could be used to develop effective regulators of mitochondrial dysfunction and novel drug candidates for various inflammatory diseases. Therefore, we believe that targeting mitochondrial dysfunction and ROS represents a promising strategy for the treatment of neurodegenerative diseases and other conditions characterized by mitochondrial dysfunction and oxidative stress. This review aims to aid in the development of effective mitochondria-targeted therapies for AD and PD, providing a comprehensive overview of current research and potential therapeutic approaches.

## Figures and Tables

**Table 1:** The compounds targeting ROS and mitochondrial dysfunction pathways in AD and PD.

Name	Target	Disease Models	Mechanism
Mitochondrial fusion and biogenesis enhancers			
SS−31,	Mitochondrial dysfunction	MPTP−injected mice, MPP^+^−treated SN4741 cells	Restores oxygen consumption rates and ATP levels, and prevents mitochondrial swelling and apoptosis [116]
		Aβ−incubated N2a cells	Prevents neurotoxicity by Increasing ATP levels, cytochrome oxidase activity, MMP, and the expression levels of MFN2, OPA1, and Prxs [117]
		APP transgenic mice (Tg2576)	Enhances PGC−1α/Nrf1/Tfam signaling−induced mitochondrial biogenesis and MFN1/2−mediated mitochondrial fusion, and suppresses H_2_O_2_ production and lipid peroxidation [118]
		APP/PS1 mice	Improves mitochondrial homeostasis by blocking DRP1 expression and inhibits apoptosis by decreasing Aβ and ROS levels [119]
BGP−15	Mitochondrial dysfunction	Primary hippocampal neurons induced by acetylated tau (TauKQ) overexpression	Reduces fragmented mitochondria and mtROS levels through the activation of PGC−1α/Nrf1/Tfam pathway and MFN1/MFN2/OPA1 signaling, preventing ATP and MMP reduction [85]
Mitochondrial fission inhibitors			
Mdivi−1	DRP1	Aβ−treated SK−NK−SH cells	Attenuates ROS levels, MMP disruption, and apoptosis by inhibiting caspase−9/−3 activation [80]
		hA53T−α-syn−induced rat	Decreases mitochondrial fragmentation, lipid peroxidation, and α-synuclein aggregates, and enhances mitochondrial respiratory capacity [120]
P110	DRP1 activity DRP1-FIS1 interation	MPP^+^−treated SH−SY5Y cells	Inhibits DRP1 translocation to the mitochondria, mitochondrial fission, and mtROS, and improves MMP and mitochondrial integrity [121]
		MPTP−injected mice	Suppresses pro-apoptotic Bax/PUMA expression by inhibiting DRP1-dependent p53 mitochondrial translocation [122]
		SH−SY5Y cells	Increases Tfam/Nrf1 expression and mitochondrial area, and decreases mitochondrial fragmentation and mtROS production [123]
Antioxidants			
CoQ_10_	ROS	Rotenone-treated HT22 cells	Prevents DRP1/FIS1−induced mitochondrial fragmentation and cell death [124]
		Aβ-injected adult rats	Protects hippocampal synaptic plasticity by decreasing serum levels of malondialdehyde and total oxidant [125]
MitoQ	mtROS	APP transgenic mice (Tg2576)	Reduces mitochondrial damage by inhibiting cyclophilin D expression and enhances neurite outgrowth [117]
		hAβ_1–42_-incubated entorhinal cortex slices	Blocks the reduction of SOD2/mitochondrial Cyt c and synaptic proteins PSD95/synaptophysin expression [126]
		6-OHDA-treated SN4741 cells and mice	Promotes MFN2−dependent mitochondrial fusion by activating PGC−1α, resulting in the suppression of mitochondrial fragmentation and neuronal apoptosis [127]
Mitochondria-driven inflammation inhibitors			
MCC950	ATPase activity of NLRP3	6-OHDA administered mice, MitoPark mice, PFF-injected mice	Inhibits the activation and release of caspase−1/IL−1β/ASC, α-synuclein accumulation, and dopaminergic degeneration [112]
OLT1177	ATPase activity of NLRP3	APP/PS1 mice	Reduces the levels of proinflammatory cytokines IL−1β, IL−6, and TNF−α, microglia activation, and the number of Aβ plaques [87]
		MPTP-injected mice	Induces the clearance of α-synuclein by increasing LC3−mediate autophagy and suppresses the levels of IL−1β, IL−18, IL−6, and IL−17A and microgliosis reactivity [113]
VX−765	Caspase−1 activity	APP^Sw/Ind^ mutant J20 mice	Decreases Iba1−positive microglial inflammation and cleaved IL−1β expression, but does not affect Aβ accumulation [89]
TDI−6570	cGAS	P301S tau transgenic mice	Restores synaptic integrity and plasticity by reducing IFN−stimulated gene expression and enhancing MEF2C transcriptional network [90]
H−151	STING	5×FAD mice	Suppresses the activation of the TBK1/p65/IRF3 pathway, the expression of neuroinflammatory genes, and Aβ_42_ levels [88]

## Data Availability

Data are contained within the article.
